# Mutations that Separate the Functions of the Proofreading Subunit of the *Escherichia coli* Replicase

**DOI:** 10.1534/g3.115.017285

**Published:** 2015-04-15

**Authors:** Zakiya Whatley, Kenneth N. Kreuzer

**Affiliations:** * University Program in Genetics and Genomics, Duke University, Durham, North Carolina 27705; † Department of Biochemistry, Duke University Medical Center, Durham, North Carolina 27710

**Keywords:** DNA polymerase, epsilon subunit, linker-scanning mutagenesis, mutation rate, SOS response

## Abstract

The *dnaQ* gene of *Escherichia coli* encodes the ε subunit of DNA polymerase III, which provides the 3′ → 5′ exonuclease proofreading activity of the replicative polymerase. Prior studies have shown that loss of ε leads to high mutation frequency, partially constitutive SOS, and poor growth. In addition, a previous study from our laboratory identified *dnaQ* knockout mutants in a screen for mutants specifically defective in the SOS response after quinolone (nalidixic acid) treatment. To explain these results, we propose a model whereby, in addition to proofreading, ε plays a distinct role in replisome disassembly and/or processing of stalled replication forks. To explore this model, we generated a pentapeptide insertion mutant library of the *dnaQ* gene, along with site-directed mutants, and screened for separation of function mutants. We report the identification of separation of function mutants from this screen, showing that proofreading function can be uncoupled from SOS phenotypes (partially constitutive SOS and the nalidixic acid SOS defect). Surprisingly, the two SOS phenotypes also appear to be separable from each other. These findings support the hypothesis that ε has additional roles aside from proofreading. Identification of these mutants, especially those with normal proofreading but SOS phenotype(s), also facilitates the study of the role of ε in SOS processes without the confounding results of high mutator activity associated with *dnaQ* knockout mutants.

The product of *dnaQ*, the ε subunit of *Escherichia coli* replicative polymerase, DNA polymerase III (Pol III), provides the 3′ → 5′ exonuclease activity for proofreading function (Echols *et al.* 1983; Scheuermann *et al.* 1983). The protein physically interacts with the α and θ subunits, gene products of *dnaE* and *holE*, respectively; together, these three subunits comprise the Pol III core. The polymerase activity is in α, whereas the function of θ has not been clearly defined beyond binding to ε (Studwell-Vaughan and O’Donnell 1993; Slater *et al.* 1994). In addition to fidelity, ε adds structural integrity, increasing the processivity of Pol III in biochemical studies (Studwell and O’Donnell 1990).

ε is a 243-amino-acid protein comprising the N-terminal proofreading domain and the C-terminal segment (εCTS) that binds to the α subunit (Figure 1A). The N-terminal domain (1–186; N186) contains three conserved exo motifs: exo I (8-21), exo II (95-108), and exo IIIε (128-192) (Bernad *et al.* 1989; Blanco *et al.* 1992; Barnes *et al.* 1995). Mutational analyses have shown that exo I, exo II, and exo IIIε contain conserved residues that are required for exonuclease activity, and the N186 domain is sufficient for proofreading (Taft-Benz and Schaaper 1998; Perrino *et al.* 1999). In addition to proofreading function, N186 also contains residues responsible for binding to θ (Derose *et al.* 2003; Keniry *et al.* 2006). The proposed Q-linker (190-212) of εCTS contains four consecutive glutamines (194-197) and remains flexible even when bound to α, as determined by NMR spectroscopy (Wootton and Drummond 1989; Ozawa *et al.* 2008). At the junction of the N-terminal domain and the C-terminal segment, there is a β-clamp binding motif (QTSMAF at amino acids 182–187) (Jergic *et al.* 2013). The β-clamp binding motif is responsible for the ε–β interaction of the tripartite complex comprising α, ε, and β (Toste Rego *et al.* 2013). Additionally, there is a poorly mapped site for chaperone DnaK binding within the εCTS (Bressanin *et al.* 2009).

**Figure 1:**
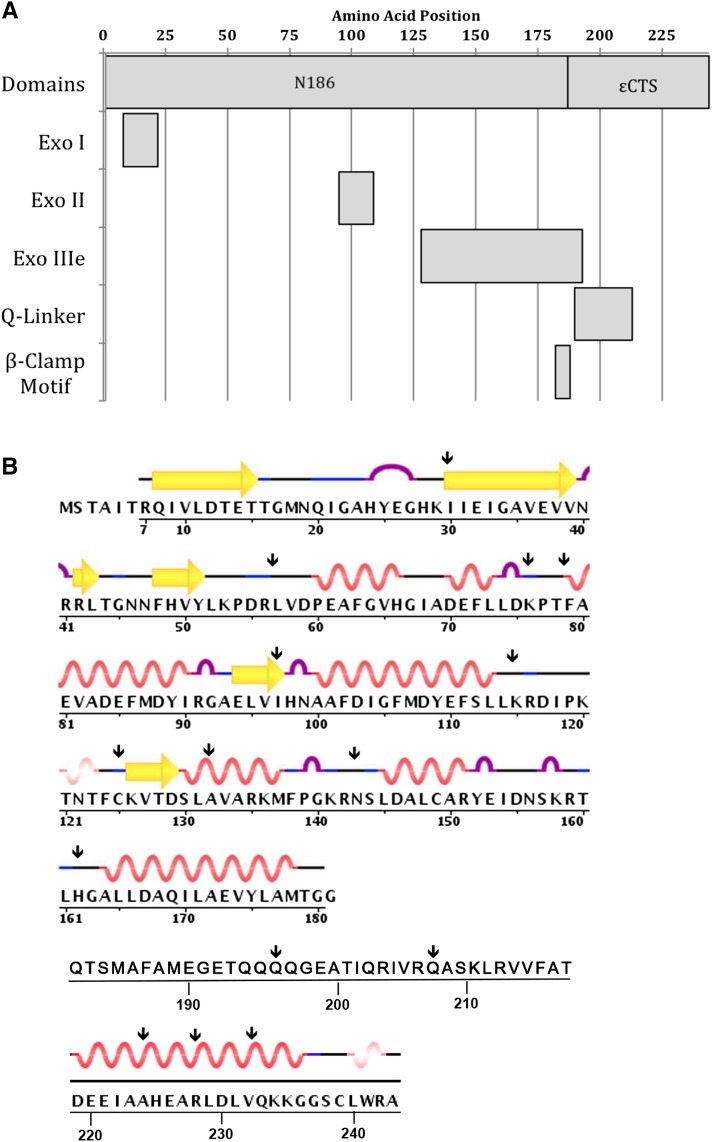
(A) Organization of ε domains and motifs. (B) Structural data from RCSB Protein Data Bank accession numbers IJ54 and 4GX8. Residues 182–218 do not have structural information available.

Various *dnaQ* mutant alleles have been discovered or created, many of which, including *dnaQ49* and *mutD5*, generate an extremely high mutator phenotype (Degnen and Cox 1974; Horiuchi *et al.* 1978; Echols *et al.* 1983; Fijalkowska and Schaaper 1996). *MutD5* (T15I) is an extreme mutator, with mutation frequencies up to 3000-fold higher than wild-type levels using rifampicin resistance as a measure (Degnen and Cox 1974; Schaaper 1988; Fijalkowska and Schaaper 1996). *DnaQ49* (V96G) is a recessive, temperature-sensitive mutant with negligible mutator activity at 28–30° but high mutator activity at 37° (Horiuchi *et al.* 1978; Echols *et al.* 1983). The ε proteins encoded by these two mutants differ in their ability to associate with α and, hence, the Pol III core (Takano *et al.* 1986). Cells carrying extreme mutator alleles of *dnaQ* are not viable, at least in part because of loss of proofreading, which leads to massive misincorporation and overload of the mismatch repair system (Schaaper 1989; Schaaper and Radman 1989; Fijalkowska and Schaaper 1996). However, the extreme increase in mutation frequency facilitates the rapid acquisition of suppressor mutations in *dnaE* (encodes α), which alleviate the growth defect and reduce the mutator phenotype (Lancy *et al.* 1989; Schaaper and Cornacchio 1992; Fijalkowska and Schaaper 1995).

In addition to the mutator phenotype, *dnaQ* mutants also display a partially constitutive SOS phenotype and an increased frequency of direct repeat recombination (Lifsics *et al.* 1992; Slater *et al.* 1994; Saveson and Lovett 1997). Although these phenotypes can be rationalized as being caused by loss of proofreading function, they may instead (or in addition) relate to the effects of ε on the behavior (*e.g.*, processivity) of DNA polymerase (for example, see discussion by Saveson and Lovett 1997).

Previous studies in our laboratory uncovered an unexpected connection between ε and quinolone-induced DNA damage in *E. coli*. Quinolones are a broad-spectrum antibiotic class that target type II topoisomerases and lead to inhibition of DNA synthesis. There are numerous quinolone derivatives, including members of the more potent subclass known as fluoroquinolones. There are also variations on the detailed mechanism of drug action, depending on the particular structure of the quinolone derivative and the cellular conditions (Drlica *et al.* 2008). Quinolones, including the parent compound nalidixic acid, target DNA gyrase in *E. coli*, stabilizing the so-called cleavage complex in which gyrase is covalently attached to the two newly cleaved 5′ ends of DNA in the strand-passage reaction intermediate. These stabilized cleavage complexes (SCCs) are reversible and their presence is necessary, but not sufficient, for cell death. Genetic evidence implies that quinolone cytotoxicity depends on formation of chromosomal double-strand breaks; however, the mechanism(s) of break formation is still unclear and the cytotoxic breaks may not be the breaks within the SCC (for review, see Drlica *et al.* 2008). The strongest evidence relates to the importance of RecBCD protein, which requires DNA ends to act. RecBC knockout mutants are hypersensitive to nalidixic acid treatment (McDaniel *et al.* 1978), implying that RecBC-dependent recombination rescues quinolone-induced breaks. Also, SOS induction following nalidixic acid treatment requires RecBCD enzyme, which processes broken ends and facilitates RecA loading for induction of the damage response (McPartland *et al.* 1980). Direct evidence of chromosome fragmentation after quinolone treatment was uncovered using sedimentation analysis and supercoiling assays (Steck and Drlica 1984).

A transposon insertion screen to identify mutants defective in SOS induction following nalidixic treatment returned 18 mutants, all of which were *recB* or *recC* mutants (Newmark *et al.* 2005). This study confirmed the RecBC-dependent nature of quinolone-induced SOS response, but failed to uncover any novel mutants. However, this screen would likely have missed mutants that were partially SOS constitutive. A subsequent screen of SOS constitutive transposon mutants revealed *dnaQ* mutants with a substantial but incomplete defect in SOS induction following quinolone treatment (Pohlhaus *et al.* 2008). This was not a general SOS defect, because the mutants were capable of RecFOR-dependent SOS induction (after treatment with mitomycin C). The *dnaQ* transposon insertion mutants therefore exhibited an SOS profile similar to *recBC* mutants, implicating ε in the mechanism of double-strand break formation after nalidixic acid treatment. Our hypothesis is that this ε role in double-strand break formation is not related to exonuclease activity, but rather reflects some function of ε in replication fork stability that is critical for how the fork responds to the quinolone-induced cleavage complexes, which have been shown to block replication forks *in vivo* (Pohlhaus and Kreuzer 2005).

The hypothesis above predicts that ε performs multiple functions, which should be mutationally separable. Isolation of separation of function mutants would substantiate the hypothesis and could also provide a very valuable reagent—a non-mutator (stable) *dnaQ* mutant with SOS phenotypes. In this study, we introduced small perturbations throughout the *dnaQ* sequence using pentapeptide linker-scanning mutagenesis. This procedure introduces variable five-amino acid cassettes, as well as a subset of small insertions with stop codons, randomly throughout the sequence of a gene, and has proven effective in the separation of phenotypes for multi-functional proteins XerD and McrA (Cao *et al.* 1997; Anton and Raleigh 2004). Five-amino-acid insertions anywhere in the coding sequence can potentially cause separation of function, whereas stop codon insertions toward the C terminus might be expected to create partially functional truncation proteins. Our results show that the proofreading function of ε can be uncoupled from the SOS phenotypes. We also isolated insertion mutants that separate partially constitutive SOS and defective nalidixic acid–induced SOS phenotypes, as well as a mutationally stable *dnaQ* allele with SOS phenotypes. Overall, our results implicate ε-dependent replisome behavior in the bacterial response to nalidixic acid.

## Materials and Methods

### Bacterial strains and plasmids

*E. coli* strain JH39 [F- *sfiA11*, *thr-1*, *leu-6*, *hisG4*, *argE3*, *ilv(Ts)*, *galK2*, *srlD*, *rpsL31*, *lacΔU169*, *dinD1*::MudI(Ap^r^ lac)] was the parental strain used to generate *dnaQ* mutants with chromosomal *dnaQ* mutations. The *dnaQ* deletion from the Keio collection (Baba *et al.* 2006) was moved into JH39 by phage P1-mediated transduction, followed by PCR verification. All plasmid constructions were done in DH5α [F^−^ *endA1 glnV44 thi-1 recA1 relA1 gyrA96 deoR nupG Φ80dlacZΔM15 Δ(lacZYA-argF)U169*, *hsdR17(r_K_^-^ m_K_^+^)*, *λ*^−^]. Plasmids are summarized in Table 1.

**Table 1:** Plasmid constructs

Plasmid	Relevant Genotype (Derivation)	Source/Reference
**pMM5**	*E. coli* chromosomal *dnaQ+*/*rnhA*+ -containing *Eco*RI fragment inserted into *Eco*RI site of pBR322	Maki *et al.* 1983
**pMM5Z**	pMM5; *Aat*II restriction site replaced with *Bgl*II using primers *Bgl*II #1(5′-TGAGATCTCGCAA CGT-3′) & *Bgl*II #2(5′-TGCGAGATCTCAACGT-3′)	This work
**Primary library**	GPS-LS Mutagenesis of pMM5Z	This work
**Secondary library**	Primary library subcloned into fresh pMM5Z backbone, removing clones with insertions outside the region of interest (*Pst*I-*Dra*III)	This work
**Final library**	Secondary library with majority of transposon excised, leaving behind a 15-bp insertion	This work
**pMAK705**	*Rep(ts)*, *cam^R^*, M13mp13 polylinker insert	Hamilton *et al.* 1989

### Linker scanning mutagenesis

Mutagenesis was performed with the New England Biolabs GPS-LS system according to provided instructions. Plasmid pMM5Z was created by digesting pMM5 with *Aat*II restriction enzyme and annealing oligos *Bgl*II #1 (5′-TGAGATCTCCGCAACGT-3′) and *Bgl*II #2 (5′-TGCGAGATCTCAACGT-3′). The transposon reaction of plasmid pMM5Z (see Table 1) was electroporated into DH5α using a BioRad Gene Pulser according to the manufacturer instructions, selecting for insertion mutants on plates with chloramphenicol and ampicillin. After overnight incubation at 37°, 1017 colonies were collected and pooled into the primary mutant library.

### Generating the *dnaQ* mutant library

Plasmid DNA from the primary library of 1017 colonies (see Table 1) was digested with *Pst*I and *Dra*III. Fragments containing the *dnaQ* gene along with the 1376-bp transposon insertion were separated by gel electrophoresis and extracted. The extracted fragments were ligated to fresh *Pst*I/*Dra*III-cleaved pMM5Z vector and electroporated into DH5α (selecting again with chloramphenicol and ampicillin). The colonies were pooled and a secondary plasmid library was generated and extracted. The secondary plasmid library was digested with PmeI, gel-purified, and re-ligated—these steps remove the majority of the transposon, leaving behind a 15-bp insertion with a single PmeI site. The desired products were isolated by transformation into DH5α. The plasmid library was harvested and then the final *dnaQ* insertion mutant library was subcloned into the polylinker of pMAK705 using *Cla*I and *Pst*I sites that flank the *dnaQ* gene.

### Site-directed mutagenesis

The Stratagene QuikChange Mutagenesis kit was used according to manufacturer’s instructions to generate the moderate mutator *dnaQ928*, which causes the G17S substitution (Taft-Benz and Schaaper 1998) in the context of plasmid pMM5Z. Similarly, we created strong (QLSLPL) and weak (ATSMAF; Q182A) β-clamp binding mutants in the motif (QTSMAF at residues 182–187) using primers QLSLPL (5′-CTG GCGATGACCGGTGGTCAACTATCGTTGCCTTTAGCGATGGAAGGAGAGAC-3′), QLSLPL_anti (5′-GTCTCTCCTTCCATCGCTAAAGGCAACGATAGTTGACCACCGGTCA TCGCCAG-3′), Q182A (5′-GGCGATGACCGGTGGTGCAACGTCGATGGCTTTT-3′), and Q182A_anti (5′-AAAAGCCATCGACGTTGCACCACCGGTCATCGCC-3′).

### Gene replacement

Following the method of Hamilton *et al.* (1989), we incorporated insertion and site-directed mutations into the chromosome of JH39 at the native location of *dnaQ*. This method uses plasmid pMAK705, with its temperature-sensitive replicon, to facilitate incorporation into the chromosome. Plasmid pMAK705 containing a *dnaQ* allele can integrate into the *dnaQ* region of the chromosome by homologous recombination, and integrants are selected on chloramphenicol plates at the nonpermissive temperature (44°). Selection is then relieved by growth at the permissive temperature (30°), which favors segregants that lose the plasmid from the chromosome. Chloramphenicol-sensitive segregants were screened for those with the desired *dnaQ* in the chromosome via PCR amplification using chromosome-specific primers dnaQ5F (5′- TGCCCCAAAACGAAGGCAGT -3′) and dnaQI3R2 (5′- TGCAGCCACAAAACGCAGTG -3′). PCR products were digested with PmeI and sequenced using primers dnaQseq1 (5′-ACCGCTCCGCGTTGTGTTCC-3′) or dnaQseq2 (5′-CGGTTGTTGGTGGTGCGGGT-3′).

### Quantitative β-galactosidase assay

The convenient *dinD*::*lacZ* reporter allows monitoring of SOS in the JH39 strain. SOS levels were determined using a β-galactosidase protocol modified for 96-well microtiter plates (Thibodeau *et al.* 2004). Overnight cultures were diluted 1:100 in LB and grown for 2 hr. They were then treated with nalidixic acid (10 μg/ml) or no drug and incubated for an additional 2 hr. Cell density (A_630_) was measured and 100 μl of each culture was lysed with Novagen PopCulture reagent at room temperature. A 15-μl aliquot of lysed cells was incubated with 135 μl Z-Buffer (40 mM Na_2_HPO_4_, 60 mM NaH_2_PO_4_, 10 mM KCl, 1 mM MgSO_4_), 0.5 μl TCEP [Tris (2-carboxyethyl) phosphine], and 30 μl of 2-Nitrophenyl β-D-galactopyranoside (4 mg/mL). The accumulation of 2-nitrophenol product was measured at A_405_ for 60 min. Miller units were calculated using the modified equation (V*1000*CF1*CF2)/(A_595_*CF3*relative volume of cell lysate used). CF1, CF2, and CF3 are conversion factors that account for absence of Na_2_CO_3_, A_415_ to A_420_, and A_595_ to A_600_, respectively (making this equation comparable to that of Miller). This method assesses the rate of β-galactosidase accumulation over time and is therefore more accurate than simple endpoint measurement β-galactosidase assays.

### Mutation rate measurements

The rifampicin-resistant colony-forming units of at least eight independent overnight cultures and the total colony-forming units of at least three overnight cultures were measured. The mutation rate of each strain was calculated using the Lea Coulson Method of the Medians Fluctuation Analysis Calculator (FALCOR) program (Foster 2006).

## Results and Discussion

### Generation of *dnaQ* mutants

To determine if the multiple phenotypes of a *dnaQ* mutant are separable, we used the GPS-LS Mutagenesis kit (NEB) to create a plasmid library of *dnaQ* insertion mutants. This system introduces a 1376-bp transposon randomly throughout the target sequence. Subsequent digestion with PmeI excises the majority of the transposon, leaving behind a 15-bp segment that encodes a 5-codon insertion and, in a subset of cases, one of the five codons is a stop codon that generates truncations in the protein.

Our general strategy involved sequencing the sites of insertion in a number of plasmid-borne *dnaQ* alleles, and then selecting a subset to move into the chromosome and replace the native *dnaQ* gene (Table 2). The subset was selected based on available structural, biochemical, and genetic data in the literature. We included insertions within both the N186 domain and the CTS. Insertions within the N-terminal domain were selected based on structural considerations (Protein Database ID: 2XY8 and 2GUI) (Berman *et al.* 2000); we chose locations that seemed less likely to greatly disrupt protein structure and proofreading/exonuclease function. Thus, we avoided highly conserved residues and chose targets on the outer surfaces of the protein rather than buried in the core. We also included two insertion/truncations, one of which (at residue 132) likely generates a complete knockout as a reference for loss of function. The other (at residue 224) creates a truncation in the CTS, which might possibly compromise a function involved in replication fork behavior without affecting proofreading function.

**Table 2:** DnaQ insertion mutants

Amino Acid Position	Insertion Composition	5-bp Target Sequence
30	IICLN	TCATT
57	LVFKQ	GCTGG
76	CLNNK	ATAAG
79	CLNTF	CGTTT
97	ILFKQ	GATCC
115	KLFKH	TAAGC
125	LFKHF	TTTCT
132	V*	CTTGC
143	NSCLN	GTTGA
162	LFKQL	GCTGC
196	LFKQQ	GCAAC
208	VFKHQ	CCTGA
224	AHV*	GCTCA
228	CLNKA	AAGCC
232	LFKQV	GGTGC

The final 15-bp insertion duplicates a 5-bp target and adds a 10-bp sequence (TGTTTAAACA) containing restriction site *Pme*I

In addition to insertion mutants, we generated three site-specific mutants based on prior studies of *dnaQ*. First, we recreated a moderate mutator allele, *dnaQ928*. This allele behaves as a dominant mutation and alters the exo I motif (G17S), indicating that it retains the ability to associate with α (Taft-Benz and Schaaper 1998). We also regenerated two interesting mutations that have been shown to effect binding of β-clamp *in vitro* (Jergic *et al.* 2013). These authors characterized a β-clamp binding motif within ε, QTSMAF, at position 182–187, and generated strong (QLSLPL) and weak (ATSMAF; Q182A) binding alleles. Interestingly, they showed that a stronger β-clamp interaction with ε led to a more stable and processive Pol III core; therefore, these mutants are expected to alter replisome behavior and might affect SOS phenotypes.

The desired subset of insertion mutations (Table 2) and the site-specific mutations were moved onto the chromosome, replacing the native *dnaQ* in the correct location and with the natural sequences for gene expression and regulation. We focused on chromosomal constructs because prior experiments revealed conflicting results when *dnaQ* mutants were expressed from a plasmid in comparison to expression from the chromosome, indicating that aberrant gene expression from the plasmid was a confounding issue (data not shown). Chromosomal gene replacement was achieved using plasmid pMAK705, a temperature-sensitive plasmid with chloramphenicol resistance (Hamilton *et al.* 1989). The *dnaQ* mutant fragment was cloned into the polylinker of pMAK705 and substituted into the chromosome of parental strain JH39 (see *Materials and Methods*). Chromosomal mutant segregants that completely lack the substitution plasmid were confirmed by lack of chloramphenicol resistance and by sequencing chromosomal PCR products that contained the mutant *dnaQ* allele. Additionally, the *dnaE* gene of each insertion and site-directed mutant was sequenced in its entirety to check for possible suppressor mutations. Although the sequencing confirmed each *dnaE* gene as wild-type, we cannot exclude the possibility of suppressor mutations elsewhere in the genome.

### Phenotypes of *dnaQ* mutants

Proofreading activity of *dnaQ* was assessed using the well-established rifampicin resistance assay as a measure of mutation rate. At least eight independent cultures of each strain were grown overnight and appropriate dilutions/aliquots were plated on LB agar with and without rifampicin, and the frequency of rifampicin resistant over total colony forming units was calculated. Mutation rates were then calculated using the Lea Coulson Method of the Medians (see *Materials and Methods*) (Foster 2006). SOS phenotypes were determined using a high-throughput β-galactosidase assay in microtiter plates, measuring both constitutive SOS levels and the ability of the mutants to induce SOS following nalidixic acid treatment. All mutation and SOS measurements are summarized in Table 3.

**Table 3:** Summary of SOS values and mutation rates

Amino Acid Position	Insertion Composition	Basal SOS	Δ Nalidixic Acid SOS	*n*	Median Mutation Rate	Upper Boundary	Lower Boundary
30	IICLN	1657 ± 108**	1553 ± 204**	4	7.63E-07	1.16E-06	3.27E-07
57	LVFKQ	1022 ± 42*	2910 ± 262*	4	5.52E-08	6.36E-08	2.45E-08
76	CLNNK	827 ± 75	2233 ± 166*	3	5.90E-08	1.33E-07	3.75E-08
79	CLNTF	1220 ± 39**	915 ± 111**	6	1.56E-06	1.85E-06	6.32E-07
97	ILFKQ	1635 ± 284	113 ± 485**	4	9.05E-07	2.05E-06	1.62E-07
115	KLFKH	934 ± 11	6617 ± 1192	4	2.19E-09	4.50E-09	1.60E-09
125	LFKHF	960 ± 67	6552 ± 1620	8	4.36E-09	9.44E-09	2.46E-09
132	V*	1419 ± 199*	629 ± 364**	8	1.83E-06	2.19E-06	2.50E-07
143	NSCLN	908 ± 78	8580 ± 1756	7	6.21E-08	1.08E-07	3.71E-08
162	LFKQL	1211 ± 51**	10,327 ± 1061**	5	9.02E-09	1.12E-08	6.58E-09
196	LFKQQ	943 ± 46	5168 ± 624	9	2.51E-09	4.31E-09	1.18E-09
208	VFKHQ	1006 ± 79	−183 ± 42**	4	3.04E-09	4.94E-09	1.32E-09
224	AHV*	1146 ± 83*	1402 ± 144**	8	1.67E-06	2.51E-06	9.16E-07
228	CLNKA	1085 ± 62*	3167 ± 256*	4	3.10E-08	1.07E-07	2.10E-08
232	LFKQV	1626 ± 95**	1208 ± 249**	4	6.89E-07	8.38E-07	4.91E-07
DnaQ928	DnaQ928	768 ± 43	2655 ± 422**	6	4.74E-08	6.42E-08	3.90E-08
Q182A	Q182A	675 ± 25	1185 ± 864**	3	1.42E-08	3.02E-08	5.51E-09
QLSLPL	QLSLPL	1204 ± 23**	6944 ± 585	6	6.44E-09	1.14E-08	4.58E-09
WT	WT	863 ± 41	5921 ± 649	11	2.84E-09	3.61E-09	2.14E-09
ΔDnaQ	ΔDnaQ	1314 ± 73**	527 ± 233**	8	6.46E-07	9.79E-07	4.04E-07

*P* values are in comparison with wild-type (no drug or nalidixic acid). *P < 0.05 and **P < 0.005. Upper and lower boundary columns present 95% C.I.s of mutation rates determined using the Lea Coulson Method of Medians as described in *Materials and Methods*.

**Figure 2:**
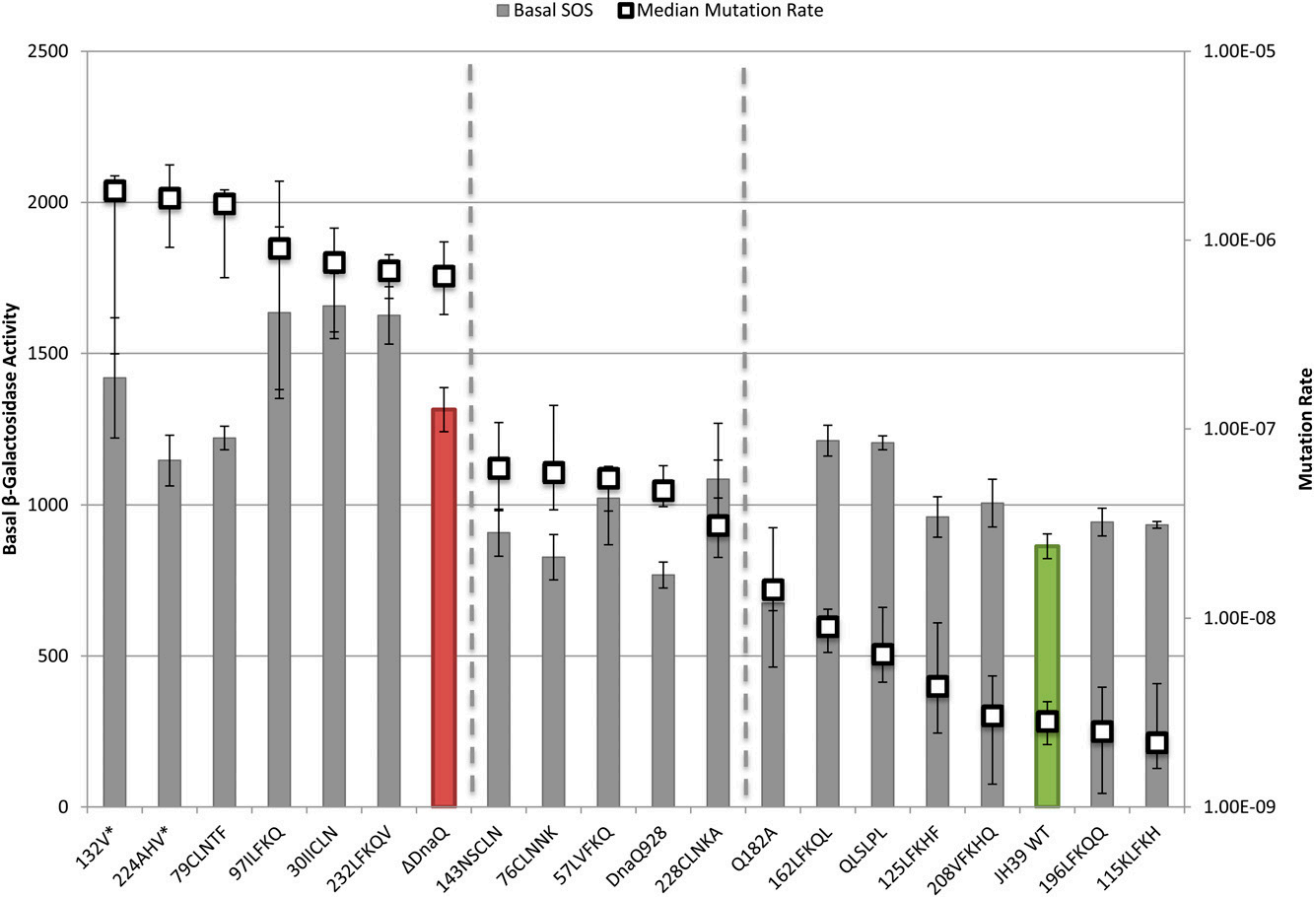
Basal SOS levels and mutation rate. SOS levels (bars) are indicated by β-galactosidase activity and correspond to the left axis. Mutation rate (□) corresponds with the right axis. Basal SOS error bars indicate SE and mutation rate error bars represent the upper and lower 95% C.I.s. Samples are sorted by decreasing mutation rate. Wild-type (WT) is green and ΔDnaQ is red.

**Figure 3:**
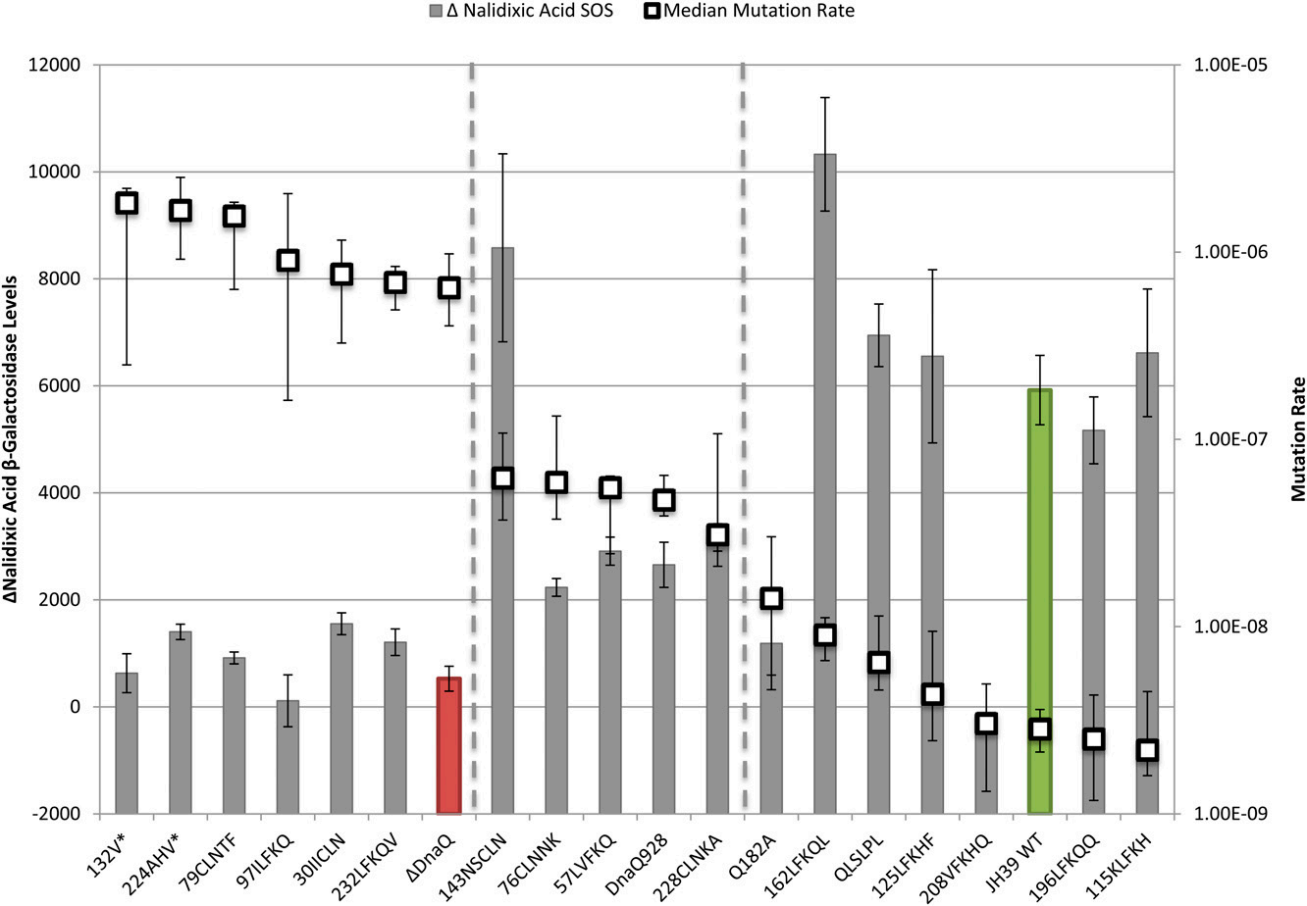
The Δ nalidixic acid SOS levels and mutation rate. The Δ nalidixic acid SOS levels (bars) represent the difference between ± nalidixic acid samples (note position of Δ nalidixic acid = 0 on the Y axis) and are indicated by β-galactosidase activity corresponding to the left axis. Mutation rate (□) corresponds with the right axis. The Δ nalidixic acid SOS error bars indicate SE and mutation rate error bars represent the upper and lower 95% C.I.s. Samples are sorted by decreasing mutation rate. Wild-type is green and ΔDnaQ is red.

**Figure 4:**
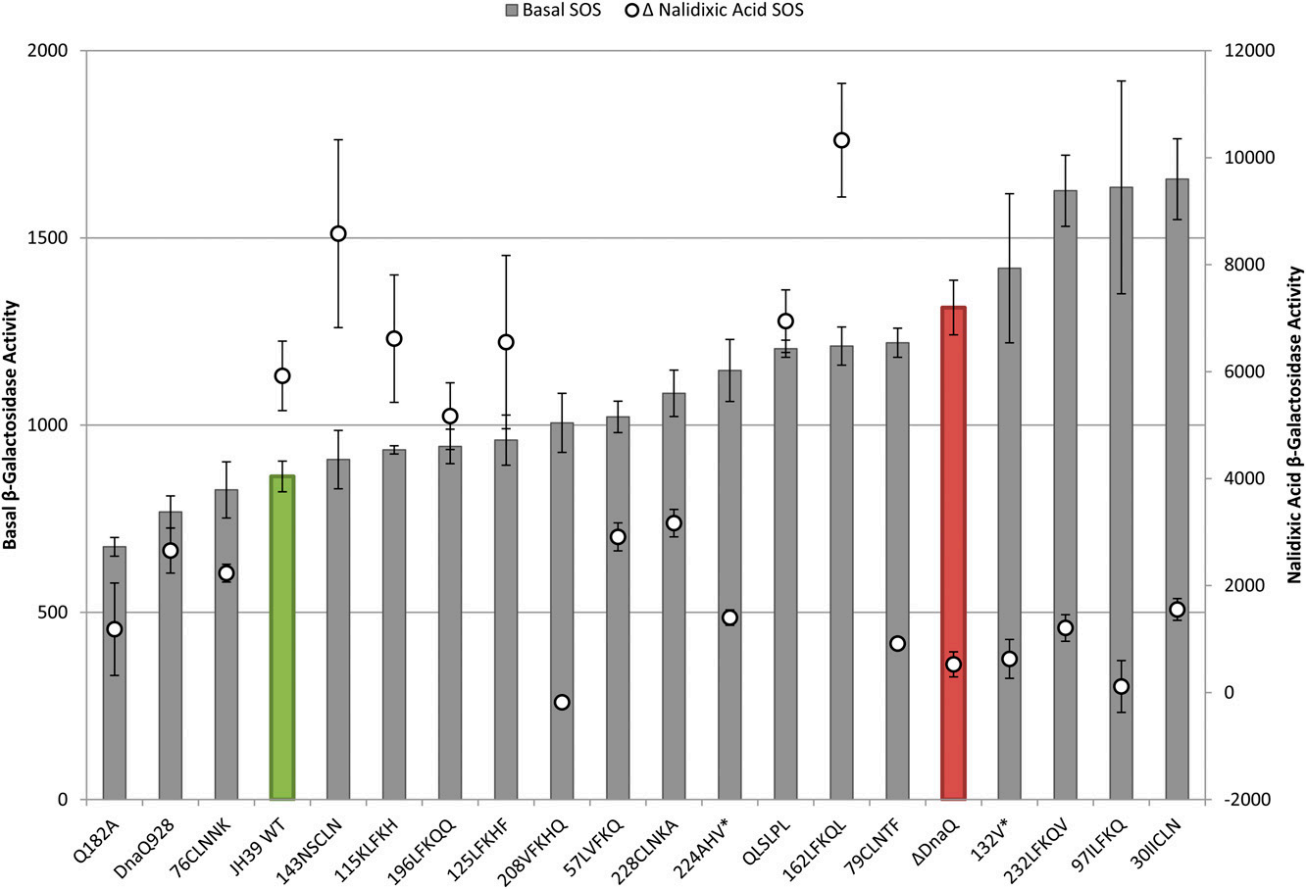
Basal and Δ nalidixic acid SOS levels. Basal β-galactosidase activity (bar) graphs to the left axis and Δ nalidixic acid values (○) to the right axis (difference between nalidixic acid SOS level and basal SOS level; note the position of 0 on Y axis). Error bars represent SE. Samples are sorted by increasing basal SOS levels. Wild-type is green and ΔDnaQ is red.

To analyze the relationships between phenotypes of the various mutants and compare them to wild-type and a *dnaQ* deletion mutant, we plotted phenotypes in pairs. First, we compared mutation rates to basal SOS levels as a measure of proofreading activity (Figure 2). The mutation rates were also compared to the increase in SOS levels with nalidixic acid treatment (ΔNalidixic acid) (Figure 3). Finally, the basal SOS levels were compared to the ΔNalidixic acid levels to look for possible separation of SOS functions (Figure 4).

In the first two comparisons, we aligned all mutants according to decreasing mutation rate from left to right (Figure 2, Figure 3). Although somewhat arbitrary, the mutants roughly fall into three categories (separated by dotted lines left to right): high mutator (like *ΔdnaQ*; red bar); moderate mutator [like *dnaQ928* (G17S)]; and non-mutator (like WT; green bar). The lowest median mutation rate of the moderate mutator group is 11-fold higher than wild-type, whereas the lowest median mutation rate in the high mutator group is more than 225-fold higher than wild-type. Three of the mutants in the right “nonmutator” category appeared to have a slight elevation (2-fold to 5-fold) of mutation rate compared to the wild-type; these include the two site-directed mutants in the β-clamp binding motif (Q182A and QLSLPL) as well as insertion mutant ^162^LFKQL.

### Insertions that behave like wild-type

Wild-type strains that are *dnaQ*^+^ are nonmutators, are not SOS constitutive, and respond to nalidixic acid treatment with high levels of SOS induction. Insertions ^115^KLFKH, ^125^LFKHF, and ^196^LFKQQ behave in this way (Figure 2, Figure 3), indicating that these insertions are innocuous for all ε functions and for important structural features of the protein. The insertion at residue 115 is at the end of α helix 4 and between motifs exo II and exo IIIε, on the outside surface of the protein. The ^125^LFKHF insertion is also between these two motifs at the end of the α helix, but before β sheet 5. Insertion ^196^LFKQQ is within the four consecutive glutamines of the Q-linker region (but does retain two runs of three glutamines). NMR studies of εCTS determined that the Q-linker region and especially the stretch of four glutamines are highly flexible; therefore, the flexible nature of this region might possibly make it tolerant to insertions or other alterations.

### Insertions that behave like *ΔdnaQ*

Complete knockout of ε leads to high mutation rate, partially constitutive SOS, and failure to induce SOS following nalidixic acid treatment. Mutants that behave in this way include ^30^IICLN, ^79^CLNTF, ^97^ILFKQ, ^224^AHV*, ^232^LFKQV, and ^132^V* (Figure 2, Figure 3). Although the insertion at 30 is not within the conserved exo I or exo II motifs, it is at the core of the protein on β-strand 2, which seems likely to disrupt protein folding (Hamdan *et al.* 2002). The truncation ^132^V* eliminates much of the exo IIIε motif and the entire C-terminal segment; again, it is not surprising that it abolishes all ε function. Insertion ^97^ILFKQ maps very close to the conditionally lethal mutator *dnaQ49* (V96G). Both mutations are within the ε exo II motif, and *dnaQ49* has been shown to be recessive and encode an ε that cannot bind to α (Takano *et al.* 1986; Jonczyk *et al.* 1998). The insertion mutant ^79^CLNTF, located between exo I and exo II, may disrupt protein stability as it is located in α helix 3, which comprises the active site along with α helix 7 and the edges of β sheets 1–3 (Hamdan *et al.* 2002).

The εCTS associates with the α subunit, and the two proteins can be cross-linked via residue 235 in the εCTS (Perrino *et al.* 1999; Taft-Benz and Schaaper 1999; Ozawa *et al.* 2008; Toste Rego *et al.* 2013). Residues 218–237 form an α helix (Ozawa *et al.* 2013), which is the site of insertion mutant ^232^LFKQV. Given the location of this insertion, outside of the proofreading NTD, we surmise that it either disrupts the ability of ε to bind to α or results in an unstable protein. A similar inference seems likely for insertion mutant ^224^AHV*, which truncates the εCTS, including the aforementioned α helix.

### Proofreading can be uncoupled from SOS phenotypes

We next turn to several apparent separations of function mutants that were uncovered. When comparing mutation rates to the basal (no drug) SOS levels, most of the mutants behaved similarly to wild-type or similarly to the ε knockout, or were somewhere in between as partially functional mutants would do (Figure 2). We found no mutants with complete separation of function, namely high mutator/low basal SOS or low mutator/partially constitutive SOS (far left and far right of Figure 2, respectively). However, a number of mutants did exhibit moderate mutator activity (reduced proofreading) yet showed wild-type levels of basal SOS expression (middle of Figure 2): G17S (*dnaQ928*); ^76^CLNNK; ^143^NSCLN; ^228^CLNKA; and ^57^LVFKQ. Although G17S and ^143^NSCLN are in motifs exo I and exo IIIε, respectively, the other insertions are not within the conserved residues related to proofreading. Insertion ^57^LVFKQ lies between motifs exo I and exo II; therefore, the behavior of this mutant aligns with a report that implicates this region for full proofreading function (Taft-Benz and Schaaper 1998). The moderate mutator phenotype of mutant ^228^CLNKA, which has an insertion within the εCTS a helix, could reflect altered binding of ε to α (see above).

Mutants with moderate mutator but low basal SOS could be explained by two models: (1) SOS is induced only by extreme levels of misincorporation or (2) the two phenotypes are caused by two different functions of ε. Further experiments are necessary to distinguish between these two models. It is worth noting that the SOS constitutive phenotype of ε knockout mutants is rather modest (*dinD*::*lacZ* expression increased by only approximately 50%; see Table 3), precluding strong conclusions based on intermediate levels of basal SOS expression.

Considering the relationship between proofreading and the ability to induce SOS after nalidixic acid treatment, many of the mutants again behaved like either wild-type or the ε knockout mutant, or somewhere in between as partially functional mutants might do (Figure 3). However, we found two striking separation of function mutants—insertion mutants ^208^VFKHQ and ^143^NSCLN (Figure 3).

Insertion mutant ^208^VFKHQ displayed fully functional proofreading but no ability to induce SOS after nalidixic acid exposure (Figure 3). This insertion is within the εCTS, outside of the proofreading domain. Because the proofreading function appears normal, the insertion presumably does not abolish the interaction of α with εCTS (although it may alter the consequences of the interaction; see *Further discussion*). Mutant Q182A has a somewhat similar phenotype to ^208^VFKHQ and is discussed below.

Insertion mutant ^143^NSCLN shows a different kind of separation of function, with a 22-fold increase in mutation rate but a robust ability to induce SOS following nalidixic acid exposure (Figure 3). This mutant has an insertion within Exo IIIε, consistent with its mutator phenotype. The ability of the ^143^NSCLN mutant to induce SOS after nalidixic acid treatment indicates that the insertion does not disrupt whatever interaction is necessary for this function (see *Further discussion*). ^143^NSCLN mutant appeared to induce somewhat higher levels of SOS with nalidixic acid treatment than the wild-type. Likewise, insertion mutant ^162^LFKQL, with a nearly wild-type mutation rate, induced SOS after nalidixic acid at a level that was significantly (74%) higher than that of the wild-type (*P* = 0.0064). These two mutants appear to generate a novel phenotype of *dnaQ* mutants hyper-responsive to nalidixic acid–induced cleavage complexes (see *Further discussion*).

### Relationship of constitutive SOS to nalidixic acid–induced SOS

We next discuss the comparison of basal SOS levels with the increase in SOS following nalidixic acid treatment; in this regard, the mutants scattered widely (Figure 4). Considering mutants with little or no ability to induce SOS following nalidixic acid treatment, phenotypes ranged from basal SOS similar to wild-type (Q^182^A, ^76^CLNNK, and ^208^VFKHQ; separation of function for SOS) to significantly higher constitutive levels than the ε knockout (^30^IICLN and ^232^LFKQV). We also identified partially constitutive mutants that are able to induce SOS following nalidixic acid treatment (^162^LFKQL and QLSLPL; discussed above and below, respectively).

### Site-directed mutants

The β-clamp binding mutants appear to be separation of function alleles. QLSLPL, the strong β-clamp binding mutant, retains catalytic proofreading function as expected but showed a basal SOS level significantly higher than wild-type (*P* = 0.0000134) and closer to the ε knockout (Figure 2; Table 3). This mutant also demonstrates separation of SOS phenotypes, because it shows a robust induction of SOS with nalidixic acid treatment (Figure 4; Table 3). This mutant ε was shown to have a stronger ε–β interaction than wild-type (Jergic *et al.* 2013), which could provide a barrier to dissociation of the replisome on encounter with endogenous damage, potentially elevating basal SOS (see *Further discussion*).

The weak β-clamp binding mutant, Q182A, revealed a particularly interesting collection of phenotypes. It demonstrated weak mutator activity (approximately five-fold higher mutation rate than that of wild-type), along with a basal SOS level that was significantly *lower* than that of wild-type (*P* = 0.0042) and greatly reduced induction of SOS following nalidixic acid treatment. These results implicate the ε-β clamp interaction in the SOS phenotypes caused by the absence of ε (see *Further discussion*).

### Further discussion

The multiple phenotypes of *dnaQ* knockout mutants are consistent with the possibility that ε has multiple functions. This possibility was raised decades ago, supported by the isolation of suppressor mutations within *dnaE* that reverse the growth deficiency but not the proofreading defect of a *dnaQ* mutant (Lancy *et al.* 1989; Lifsics *et al.* 1992). The isolation of separation of function mutants in this report strongly supports the proposal of multiple ε functions (Table 4). Certain separation of function mutants isolated here also provide a very useful reagent, namely ε variants with defects in functions relating to SOS induction but wild-type mutation rates, *i.e.*, mutationally stable strains that do not produce confounding results due to spurious global mutations. Our data also identify sites in ε (residues 115, 125, 196) that are tolerant of five amino-acid insertions with no phenotypic consequence.

**Table 4:** Summary of separation of function mutants

	SOS Constitutive	Nalidixic Acid SOS Defect	Mutator Activity
WT	No	No	No
ΔDnaQ	Yes	Yes	High
DnaQ928	No	Yes	Moderate
Q182A	No	Yes	Low
QLSLPL	Yes	No	Low
^76^CLNNK	No	Yes	Moderate
^143^NSCLN	No	No	Moderate
^162^LFKQL	Yes	No	Low
^208^VFKHQ	No	Yes	No

Yes or no indicates presence or absence of the indicated phenotype. Mutator phenotypes were characterized into four groups to reflect the wide range that was observed.

These results can be viewed through the lens of recent studies on the clamp binding motif of ε and the interactions between clamp, ε, and α (Jergic *et al.* 2013; Toste Rego *et al.* 2013). Toste Rego *et al.* (2013) analyzed the interactions of the three proteins, demonstrating that ε enhances the stability of the clamp–α complex and identifying sites where each pair of proteins can be cross-linked to each other. The increase in stability of the complex due to ε binding is presumably related to the stimulation of polymerase processivity by ε (Studwell and O’Donnell 1990; Kim and Mchenry 1996), and clamp binding by ε, in turn, improves proofreading ability (Toste Rego *et al.* 2013). Based on these and other results, Toste Rego *et al.* (2013) developed a model in which α and ε simultaneously bind to the canonical binding pocket of the two protomers of the β clamp dimer. Importantly, they also identified cross-links between α and both the proofreading NTD of ε and the extreme ε C-terminus (residue 235). These results led to an elegant model for how the polymerase complex responds to both template damage and misincorporation.

As mentioned above, misincorporated bases are more efficiently proofread by ε in the context of the tripartite complex. Template damage, however, cannot be corrected by ε, but rather is proposed to cause weakening of the clamp–ε interaction, which can allow binding of the translesion polymerase Pol IV. One important feature of the model is that ε remains associated with α via the flexible εCTS even when the ε–clamp association is lost. Several aspects of this model could relate to the phenotypes of ε mutants analyzed here, and we propose that the dynamic behavior of the tripartite complex described above could directly relate to the response of the replisome to endogenous lesions and to blocking gyrase cleavage complexes.

As the tripartite complex encounters endogenous damage, the weak clamp binder (Q^182^A) would be expected to more readily dissociate from the clamp, allowing translesion polymerases to access the free site on the β clamp. Q^182^A has weak mutator activity (five-fold higher than wild-type), and we propose two mechanisms that could lead to this. One scenario is that Q182A has weaker exonuclease activity, and this leads to less efficient proofreading, hence the weak mutator activity observed. The other scenario is that the weak binder allows Pol IV to access the β clamp more often, with or without a lesion present. Pol IV is a low-fidelity polymerase, and this occasional access could be the cause of the weak mutator activity. If the weak clamp binder allows the translesion polymerase to associate more easily, it could synthesize past the lesion and dissociate, then Q^182^A ε (still bound to α) would regain access to the clamp to continue high-fidelity replication without inducing SOS. More efficient TLS across the endogenous template damage is thus proposed to reduce basal SOS levels, which is observed for Q^182^A.

Under these same assumptions, the strong clamp binder, QLSLPL, would remain bound to the β clamp, preventing access by the translesion polymerase. This mutant would not easily allow the translesion polymerase to access the β clamp. This is consistent with the mutation rate similar to that of wild-type observed with this mutant. When facing endogenous damage, the replication fork with ε tightly bound to α would stall, because the translesion polymerase does not have access to the β clamp in QLSLPL and the damage could not be readily bypassed. This prolonged polymerase stalling could result in increased SOS induction, explaining the constitutive SOS phenotype of QLSLPL.

Our results indicate that the ε–β clamp interaction is required for nalidixic acid–induced SOS, such that the weak clamp binder leads to defective SOS induction following nalidixic acid. These results are consistent with a particular version of the replication run-off model. We know that quinolone-induced SCCs cause replication fork stalling, and stalling presumably leads to DnaB helicase unloading. We propose that the tripartite polymerase complex usually cannot proceed to the SCC at this stage, but occasionally moves forward, encountering and disrupting the SCC. The resulting broken arm of the replication fork creates a double-stranded end for RecBC to resect, which in turn is recognized by RecA for SOS induction.

As outlined above, the weak clamp mutant (Q182A) more easily allows translesion polymerases to associate with the tripartite complex. We propose that this weaker, less-processive tripartite complex would not have the ability to move forward and disrupt the SCC. Thus, the weak clamp binder does not create a break or double-strand end for RecBC, leading to the defect in nalidixic acid-induced SOS.

With the strong clamp-binding mutant (QLSLPL), the tripartite complex is tightly associated, showing even higher processivity than the wild-type complex (Jergic *et al.* 2013). According to this model, this mutant complex moves forward efficiently, readily disrupting the SCC and thereby generating double-strand ends and inducing SOS efficiently (equal to or perhaps slightly higher than wild-type; see Figure 3).

In this model, an ε knockout causes a defect in nalidixic acid–induced SOS response because ε is required for sufficient processivity to disrupt the SCC. In several of our SOS-defective insertion mutants (^30^IICLN, ^79^CLNTF, ^132^V*, ^224^AHV*, and ^232^LFKQV), the high mutators are likely knockouts due to protein instability, misfolding, and/or inability to associate with α at the tripartite complex, eliminating all functions of ε.

This replication run-off model can explain some of the striking separation of function mutants. For example, ^162^LFKQL behaves similar to the strong clamp binder; it is a low mutator (consistent with preventing Pol IV access to the clamp), SOS constitutive, and able to induce SOS after nalidixic acid at levels even higher than that of wild-type. Like the weak β-clamp mutant Q182A, ^76^CLNNK has modestly higher mutation rate and lower nalidixic acid–induced SOS levels than the wild-type (with basal SOS levels in between those of Q182A and wild-type). Biochemical characterization of these various *dnaQ* mutants could directly test whether they affect the tripartite complex as predicted and whether disruption of the SCC occurs in the manner predicted by the replication run-off model.
